# Supplementary material for: A Molecular Electron Density Theory Study of the [3+2] Cycloaddition Reaction of an Azomethine Ylide with an Electrophilic Ethylene Linked to Triazole and Ferrocene Units
Source: Molecules. 2022 Oct 3;27(19):6532. doi: 10.3390/molecules27196532 (PMC9571756; doi:10.3390/molecules27196532)
Supplement: Supplementary file 1 [file molecules-27-06532-s001.zip › molecules-1939376-supplementary.pdf]

Supplementary Material

A Molecular Electron Density Theory Study of the [3+2] Cycloaddition Reaction of an Azomethine Ylide with an Electrophilic Ethylene Linked to Triazole and Ferrocene Units

Luis R. Domingo,[1*] Mar Ríos-Gutiérrez,[1] and Assem Barakat[2]

1 Department of Organic Chemistry, University of Valencia, Dr. Moliner 50, 46100 Burjassot, Valencia, Spain
2 Department of Chemistry, College of Science, King Saud University, P. O. Box 2455, Riyadh 11451, Saudi Arabia
* Correspondence: luisrdomingo@gmail.com

Index

S2 Figure with the isosurface of **TS-mn**

S3 Table with the *ω*B97X-D/6-311G(d,p) total electronic energies, in gas phase and in methanol, of the stationary points involved in the 32CA reaction of AY **21** with ferrocene ethylene **22**.

S3 Table with the *ω*B97X-D/6-311G(d,p) thermodynamic data of the stationary points involved in the 32CA reaction of AY **21** with ferrocene ethylene **22**.

S4 *w*B97X-D/6-311G(d,p) gas phase computed total energies, unique imaginary frequency, and Cartesian coordinates of the stationary points involved in the 32CA reaction of AY **21** with ferrocene ethylene **22**.

.

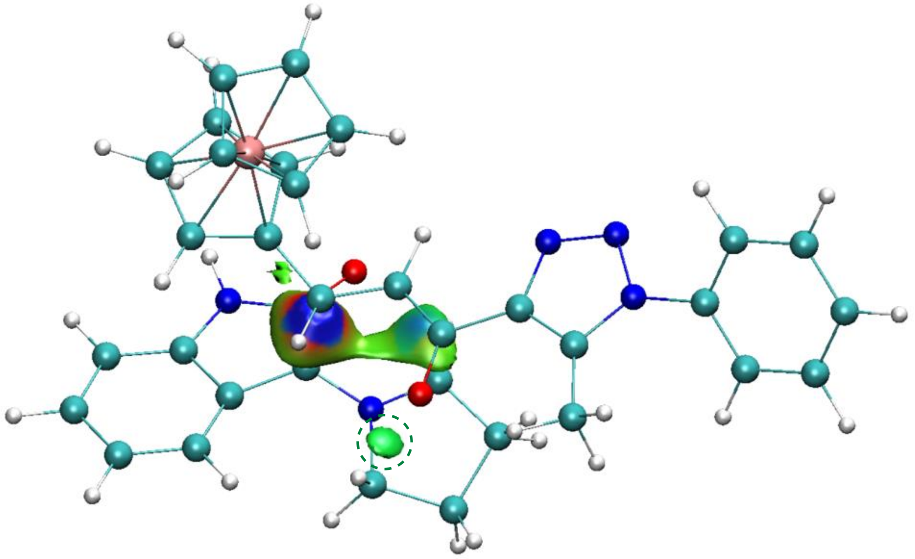

Figure S1. IGM-δg_{inter} 0.018 a.u. isosurface of **TS-mn** represented with a blue-green-red color-code in the range -0.08 < (sign) $\lambda_2\rho$ < 0.08 a.u. The attractive NCI surface associated with the hydrogen bond is highlighted by a line-dashed green circle.

Table S1. ωB97X-D/6-311G(d,p) total electronic energies, in au, in gas phase and in methanol, of the stationary points involved in the 32CA reaction of AY **21** with ferrocene ethylene **22**.

	gas phase	*methanol*
AY **21**	-649.1589842	-649.1751303
ethylene **22**	-2352.833218	-2352.848514
**MC-on**	-3002.032996	-3002.056543
**TS-on**	-3002.019461	-3002.041514
**TS-ox**	-3002.006151	-3002.033624
**TS-mn**	-3002.010928	-3002.035144
**TS-mx**	-3002.002885	-3002.028317
**23**	-3002.071765	-3002.095599
**24**	-3002.062666	-3002.087048
**25**	-3002.074949	-3002.096978
**26**	-3002.079989	-3002.102857

Table S2. ωB97X-D/6-311G(d,p) enthalpies (H, in au), entropies (S, $cal \cdot mol^{-1} \cdot K^{-1}$) and Gibbs free energies (G, in au), computed at 65 ºC in methanol, of the stationary points involved in the 32CA reaction of AY **21** with ferrocene ethylene **22**.

	H	S	G
AY **21**	-648.936608	113.2	-648.997552
ethylene **22**	-2352.454592	176.8	-2352.549753
**MC-on**	-3001.421216	241.6	-3001.551264
**TS-on**	-3001.407360	230.2	-3001.531263
**TS-ox**	-3001.399409	231.9	-3001.524250
**TS-mn**	-3001.401385	239.7	-3001.530420
**TS-mx**	-3001.394489	242.4	-3001.524987
**23**	-3001.458237	231.8	-3001.583014
**24**	-3001.449450	230.6	-3001.573578
**25**	-3001.459185	235.3	-3001.585865
**26**	-3001.465555	229.8	-3001.589259

*w*B97X-D/6-311G(d,p) gas phase computed total energies, unique imaginary frequency, and Cartesian coordinates of the stationary points involved in the 32CA reaction of AY **21** with ferrocene ethylene **22**.

AY **21**
E = -649.1589842 a.u.

C	-2.64364000	0.64395300	-0.10252900
N	-1.54121000	-0.05679300	-0.03617600
C	-0.25158900	0.40707700	-0.02185000
C	0.99368300	-0.31945800	-0.01800700
C	2.00679100	0.67430000	0.03707900
C	3.35309500	0.35953900	0.05036600
C	3.72072600	-0.98279700	0.00213400
C	2.74909900	-1.97523400	-0.06591600
C	1.39381200	-1.65618100	-0.08033300
C	0.02051000	1.82098600	0.01427100
H	4.10255600	1.14212200	0.09460100
H	0.67254200	-2.46019200	-0.15106100
C	-1.82253800	-1.50978800	-0.00314500
H	-2.57342400	1.72104000	-0.15853700
H	-1.20821300	-1.96888900	0.76915500
N	1.40658100	1.91922300	0.06150700
H	-1.54046600	-1.92592600	-0.97321000
O	-0.75356900	2.77629100	0.01484900
H	1.87951800	2.80509200	0.08888500
H	4.77049500	-1.25154600	0.01225700
H	3.04746100	-3.01636500	-0.11275800
C	-3.86054400	-0.21533300	-0.14752600
H	-4.65213800	0.15409100	0.50816500
H	-4.27424500	-0.23552100	-1.16418400
C	-3.32193500	-1.59002800	0.28278400
H	-3.48560600	-1.73473000	1.35218900
H	-3.79235500	-2.41872400	-0.24559300

ferrocene ethylene **22**
E = -2352.833218 a.u.

C	-0.48712300	0.90866900	-0.44802100
C	-1.67190100	1.43330000	-0.11472000
C	0.72698100	1.29709500	0.29465300
H	-1.68860600	2.14972700	0.70368500
H	-0.37392000	0.18985600	-1.25057500
O	0.72387600	2.11324400	1.20192600
C	1.98185800	0.63128900	-0.10896800
N	4.00279800	-0.06188200	-0.30000200
N	3.24889700	-0.72515800	-1.21558100
C	3.24658200	0.79674300	0.42401400
C	3.74176300	1.64560900	1.54049500

H	4.10139600	2.60799400	1.16605600
H	2.91534500	1.85065700	2.21777400
H	4.55938800	1.15719900	2.07407500
C	5.38946300	-0.36024400	-0.19028400
C	5.78818200	-1.69141000	-0.16876900
C	6.32230400	0.66701400	-0.12241100
C	7.13887200	-1.99222300	-0.07141000
H	5.03960200	-2.46982100	-0.23947700
C	7.67049900	0.35407200	-0.00955600
H	5.99804100	1.69884000	-0.17453400
C	8.08043300	-0.97296700	0.01618800
H	7.45572800	-3.02827500	-0.05770100
H	8.40164500	1.15181500	0.04515000
H	9.13385500	-1.21318600	0.09854700
N	2.04462400	-0.30559900	-1.09725100
C	-5.50603800	-1.72255500	0.71208100
C	-5.22892300	-0.74761800	1.70909100
C	-3.82557500	-0.73583100	1.93276100
C	-3.23459400	-1.70234000	1.07390400
H	-4.14492200	-3.06618500	-0.44077500
H	-6.47766900	-1.95193500	0.30148000
H	-5.95294900	-0.10514900	2.18685400
H	-3.29382800	-0.08067700	2.60623300
H	-2.17748800	-1.90010200	0.97770600
C	-4.27350900	-2.31374900	0.32252200
C	-5.21995000	1.11829200	-1.14809700
C	-4.19748900	1.72074900	-0.37366800
C	-2.94780500	1.12667600	-0.73879400
C	-3.22100000	0.14743500	-1.74537300
H	-5.13448100	-0.50233700	-2.68361200
H	-6.27573500	1.33294700	-1.08274500
H	-4.33009600	2.47990400	0.38308000
H	-2.49378800	-0.50034800	-2.21091400
C	-4.61599500	0.14809000	-1.99560200
Fe	-4.23342200	-0.29211400	-0.02638800

MC-on

E = -3002.032996 a.u.

C	-1.75960700	3.09384900	0.32170000
N	-0.48146300	3.01699000	0.08370500
C	0.46916100	2.38818200	0.85367800
C	-1.00893900	-0.10427700	-0.50955200
C	-2.15772500	0.32453400	-1.04400000
C	0.25024700	0.01337000	-1.26215200
H	-2.13028700	0.69335100	-2.06743800
H	-0.96658900	-0.53403500	0.48314900
O	0.34638500	0.66220600	-2.29804500
C	1.89342700	2.30740100	0.66488600

C	2.36354100	1.46723400	1.70952000
C	3.69635100	1.13167900	1.84682700
C	4.60782300	1.63699200	0.92223800
C	4.17552400	2.47740700	-0.09814500
C	2.83174500	2.81997800	-0.23243700
C	0.10021500	1.61668600	2.01042900
H	4.01978200	0.49315900	2.66114100
H	2.53969400	3.48416700	-1.03528800
C	-0.12474600	3.64646200	-1.20891700
H	-2.15111200	2.67271800	1.23656200
H	0.52890700	2.96936200	-1.75392900
N	1.29238400	1.09511100	2.49509300
H	0.39884500	4.58089000	-0.99208500
O	-1.00819800	1.40460400	2.50809200
H	1.30786000	0.39051000	3.21146200
H	5.65655900	1.37660700	1.00605100
H	4.89505300	2.87794300	-0.80367300
C	-2.50460200	3.79007200	-0.76188500
H	-3.41322400	3.24417400	-1.02844600
H	-2.81570300	4.78846300	-0.42969000
C	1.41448400	-0.69134900	-0.70447200
N	3.35996400	-1.48506400	-0.27624000
N	2.50930500	-1.92427900	0.68901300
C	2.71706100	-0.69598500	-1.16718800
C	3.34524300	-0.00550700	-2.32336700
H	3.34266900	-0.64799600	-3.20862500
H	2.75815900	0.87954000	-2.55598800
H	4.37193100	0.28202300	-2.09358700
C	4.73140100	-1.85141500	-0.21035400
C	5.42116100	-2.20494600	-1.36330200
C	5.36164800	-1.87271700	1.02865100
C	6.76393900	-2.54947900	-1.27628600
H	4.91151000	-2.22859400	-2.31763200
C	6.69786900	-2.23378800	1.10632700
H	4.79241100	-1.61705300	1.91120800
C	7.40543600	-2.56207700	-0.04498400
H	7.30387400	-2.82206500	-2.17532400
H	7.19009700	-2.25297300	2.07151200
H	8.45185200	-2.83603000	0.01953200
C	-1.47322000	3.84408000	-1.90203900
H	-1.63676500	3.01861100	-2.59596200
H	-1.50879900	4.77633800	-2.46509400
N	1.34916900	-1.45065400	0.42524800
C	-3.80722800	-2.92290400	-1.00854000
C	-5.22646300	-2.97314100	-0.94071300
C	-5.59703700	-3.05741700	0.42945900
C	-4.40599300	-3.06070000	1.20643800
H	-2.25885500	-2.91596300	0.59934200
H	-3.21520000	-2.81609200	-1.90506500
H	-5.90458700	-2.92145500	-1.77909800

H	-6.60534900	-3.08147700	0.81433300
H	-4.35122600	-3.07996600	2.28428700
C	-3.29993300	-2.97609000	0.31802300
C	-3.45051200	0.33101100	-0.37297900
C	-4.72462100	0.36583000	-1.02153600
C	-5.73106200	0.30740900	-0.02352600
C	-5.08767000	0.25002900	1.24483300
H	-2.91319200	0.24624000	1.79383600
H	-4.88394400	0.38296900	-2.09020900
H	-6.79620300	0.28061200	-0.19717800
H	-5.58173900	0.17961100	2.20202800
C	-3.68541200	0.25991700	1.03724000
Fe	-4.51755600	-1.34710700	0.08686600

TS-on
E = -3002.019461 a.u.
1 imaginary frequency -403.8442 cm-1

C	-2.05235100	2.71076800	-0.00962600
N	-0.73282800	2.97637800	-0.09209100
C	0.18745600	2.38054400	0.69065100
C	-0.97577600	0.11795400	-0.22700800
C	-2.14947900	0.70470700	-0.66400200
C	0.20779500	0.15047800	-1.05425900
H	-2.16201000	0.98304500	-1.71516700
H	-0.94644800	-0.46156600	0.68492400
O	0.27986500	0.77384900	-2.11820400
C	1.63191200	2.37490400	0.56804200
C	2.11302900	1.67764800	1.69978200
C	3.45229500	1.38542900	1.87384900
C	4.34987000	1.81552700	0.90041300
C	3.90429100	2.52507800	-0.20929200
C	2.55148100	2.80365500	-0.38607900
C	-0.15708600	1.77832300	1.99391300
H	3.78807100	0.83593500	2.74545500
H	2.23978100	3.35037900	-1.26427900
C	-0.39722700	3.73136100	-1.31866400
H	-2.44260800	2.45315100	0.96381400
H	0.34380500	3.18010700	-1.88875200
N	1.05078300	1.36215500	2.52380700
H	-0.00005600	4.70522000	-1.01899500
O	-1.23484000	1.67072200	2.55653000
H	1.09596500	0.77974000	3.34169300
H	5.40498500	1.59410700	1.01245200
H	4.61661300	2.86241800	-0.95305100
C	-2.79563900	3.55069600	-1.00336600
H	-3.67423700	3.03179700	-1.39121500
H	-3.14508900	4.47496200	-0.52881600
C	1.37830700	-0.60268800	-0.55380000

N	3.33393900	-1.40862200	-0.19096700
N	2.54096600	-1.76768900	0.84907100
C	2.64577700	-0.66968400	-1.09559300
C	3.21538700	-0.08400600	-2.33772300
H	3.19542800	-0.80958100	-3.15595400
H	2.59631400	0.76096500	-2.63006800
H	4.24611200	0.23883200	-2.18123800
C	4.70914400	-1.76364000	-0.17179800
C	5.32620400	-2.25165600	-1.31679000
C	5.41980200	-1.62799400	1.01558600
C	6.67503200	-2.58086000	-1.27689900
H	4.75412500	-2.38936500	-2.22521500
C	6.76197300	-1.97521600	1.04903400
H	4.90694100	-1.26358700	1.89529700
C	7.39510400	-2.44272200	-0.09749400
H	7.15849500	-2.95984300	-2.16952400
H	7.31704500	-1.87478700	1.97432200
H	8.44579900	-2.70625200	-0.06887500
C	-1.72738300	3.82276600	-2.07160600
H	-1.75434800	3.04432400	-2.83577300
H	-1.85105900	4.78771500	-2.56288800
N	1.37284600	-1.28770500	0.62479200
C	-3.06492700	-2.78792600	-1.07975700
C	-4.44647200	-3.08770800	-1.23748700
C	-4.96612400	-3.44502500	0.03712200
C	-3.90549600	-3.36525800	0.98060300
H	-1.76004000	-2.78133100	0.72919800
H	-2.39430200	-2.44074500	-1.85165200
H	-5.00948300	-3.02282400	-2.15636500
H	-5.99247800	-3.69830400	0.25615300
H	-3.98522100	-3.54605800	2.04183900
C	-2.73023600	-2.96069500	0.28995000
C	-3.46026500	0.35683100	-0.08677500
C	-4.67771700	0.23261300	-0.82000400
C	-5.70513100	-0.15268200	0.08347700
C	-5.12956500	-0.25394300	1.37966100
H	-3.02691200	0.08548000	2.08222600
H	-4.78042600	0.35744700	-1.88859600
H	-6.73241200	-0.36102700	-0.17483600
H	-5.64558900	-0.54842100	2.28095100
C	-3.74850000	0.06011400	1.27877100
Fe	-4.19768500	-1.54254900	0.08488700

TS-ox
E = -3002.006151 a.u.
1 imaginary frequency -412.2270 cm-1

C	1.87768300	2.45985900	1.37566900
N	1.12757600	2.76709500	0.29724700

C	-0.15532600	2.35726000	0.19265000
C	0.78946000	0.00953700	0.59502600
C	2.03293200	0.35786000	1.09687500
C	-0.30331800	-0.40018700	1.45468900
H	2.18690000	0.18149600	2.15823500
H	0.64652200	-0.14947300	-0.46605800
O	-0.27370300	-0.35850300	2.67867300
C	-0.98460500	2.21152200	-0.99162200
C	-2.31682200	2.13659700	-0.53237800
C	-3.37806800	1.88168300	-1.37961800
C	-3.10345400	1.70213300	-2.73224000
C	-1.79907400	1.77263700	-3.20995400
C	-0.73613000	2.02033300	-2.34738500
C	-1.06013000	2.46578300	1.36016100
H	-4.39109100	1.81421100	-0.99891200
H	0.27113200	2.04945800	-2.74220100
C	1.94710200	3.22447000	-0.83354500
H	1.35101500	2.33585500	2.31162300
H	1.49015800	4.12234900	-1.25348600
N	-2.33442300	2.32098200	0.84010700
H	1.97013800	2.45228500	-1.60291800
O	-0.80590300	2.67401500	2.53018900
H	-3.15039300	2.31487300	1.42675000
H	-3.91719300	1.50083000	-3.41964700
H	-1.60476500	1.62081900	-4.26476800
C	3.20113000	3.15370900	1.27415000
H	3.18073100	4.07027500	1.87247500
H	4.01606300	2.53472400	1.65125800
C	-1.51980400	-0.85930300	0.74150500
N	-3.53021400	-1.30737400	0.14174600
N	-2.72185800	-1.52724400	-0.92521800
C	-2.81232000	-0.88523500	1.21466800
C	-3.36151600	-0.47160400	2.53365200
H	-3.48215200	-1.33050400	3.19886000
H	-2.64938900	0.20238700	3.01026700
H	-4.33504900	0.01330300	2.42432700
C	-4.93349300	-1.45705500	-0.01238400
C	-5.69883800	-2.02481300	0.99917700
C	-5.52258800	-1.03785600	-1.20124300
C	-7.07168100	-2.14766300	0.82736400
H	-5.22453400	-2.38416600	1.90333300
C	-6.89206800	-1.18104800	-1.36744600
H	-4.89454000	-0.62191800	-1.97820700
C	-7.67097500	-1.72607400	-0.35245700
H	-7.67039700	-2.58855500	1.61559900
H	-7.35352600	-0.86031600	-2.29407400
H	-8.74150500	-1.82947200	-0.48463700
C	3.34454700	3.45303300	-0.23262600
H	3.68933800	4.47156200	-0.41060000
H	4.06054100	2.76895000	-0.68761600

N	-1.52305100	-1.25112800	-0.56350000
C	3.22141300	-3.02637900	0.84991000
C	4.61253500	-3.25989700	0.66671800
C	4.85340400	-3.40297800	-0.72727200
C	3.61114700	-3.25704800	-1.40356600
H	1.55794600	-2.82765700	-0.62429200
H	2.72381000	-2.83425700	1.78884400
H	5.35895500	-3.29117600	1.44602100
H	5.81458400	-3.56240700	-1.19231200
H	3.46371900	-3.28343300	-2.47265300
C	2.60233000	-3.02423800	-0.42855300
C	3.23006700	0.22721400	0.24752900
C	4.57159600	0.11201700	0.71880500
C	5.43316200	-0.06094200	-0.39882500
C	4.63159100	-0.04854100	-1.57264900
H	2.42329900	0.11812400	-1.84115100
H	4.86783600	0.10481100	1.75797400
H	6.50157000	-0.20961100	-0.36004700
H	4.98345900	-0.18748200	-2.58371000
C	3.27578000	0.12244500	-1.17777900
Fe	4.03359400	-1.56306400	-0.32976500

TS-mn
E = -3002.010928 a.u.
1 imaginary frequency -339.8056 cm-1

C	0.26036300	-2.52872700	-1.20332400
N	-0.77691800	-2.77016400	-0.43523400
C	-1.95094200	-2.03480000	-0.50077500
C	-1.25991300	-0.16780100	0.11034500
C	0.05246600	-0.04890200	-0.35650200
C	1.14175500	-0.44119000	0.47808800
H	-1.34395500	-0.44524500	1.15863600
H	0.27684800	0.48902100	-1.26766600
O	1.03260300	-1.04275400	1.55999200
C	-3.13025300	-2.26878700	0.34044300
C	-4.25219000	-1.93673400	-0.43886800
C	-5.52887400	-1.89429000	0.08950900
C	-5.68506500	-2.19146400	1.44096000
C	-4.58709900	-2.50592400	2.23436600
C	-3.30323100	-2.53247600	1.69269200
C	-2.46882300	-1.71434600	-1.86102700
H	-6.37862500	-1.62402700	-0.52622500
H	-2.45474100	-2.73966700	2.33283200
C	-0.42969500	-3.62145300	0.72072600
H	0.13689000	-1.96021800	-2.11028700
H	-1.23812000	-4.32954500	0.90122200
N	-3.83904400	-1.64867900	-1.73303900
H	-0.29155400	-2.96854100	1.58338200

O	-1.84501300	-1.52838600	-2.88697000
H	-4.41956200	-1.26843000	-2.46084400
H	-6.67593500	-2.16649400	1.87947900
H	-4.72734200	-2.72174600	3.28686700
C	1.44410000	-3.34055800	-0.80001800
H	1.87109300	-3.87524000	-1.65110800
H	2.22489000	-2.68101600	-0.40825600
C	2.50475800	-0.09736600	-0.00386800
N	4.62148800	0.18958300	-0.23772000
N	4.01315100	0.68564100	-1.34309200
C	3.70963700	-0.31003900	0.63608600
C	4.02892600	-0.87861400	1.97423500
H	4.86607700	-0.35106200	2.43653300
H	3.13977400	-0.80679300	2.59680200
H	4.29335600	-1.93760100	1.90079500
C	0.88216500	-4.27749600	0.28599600
H	0.68733100	-5.26625400	-0.13448600
H	1.56311300	-4.39182700	1.12814700
N	2.75072500	0.51072700	-1.19908500
C	6.03374100	0.29253300	-0.11585200
C	6.78236800	-0.79481800	0.31791700
C	6.64974900	1.49194700	-0.45462800
C	8.16064600	-0.67072700	0.43336300
H	6.29364500	-1.73340500	0.54661600
C	8.02882700	1.59956900	-0.35116500
H	6.04309300	2.31720000	-0.80395800
C	8.78578300	0.52396200	0.09964400
H	8.74687500	-1.51635800	0.77313000
H	8.51291500	2.53158600	-0.61784500
H	9.86213800	0.61540900	0.18536600
C	-4.26806000	1.98233400	-0.35181400
C	-3.50874300	1.03999700	0.38944300
C	-2.32830300	0.73282800	-0.35078400
C	-2.37343000	1.48619400	-1.56242000
H	-3.86811400	2.95638200	-2.32366700
H	-5.19481200	2.43808000	-0.03722500
H	-3.75708100	0.63639600	1.36056100
H	-1.61830900	1.48553000	-2.33317800
C	-3.56713800	2.25536600	-1.55959300
C	-2.45725600	4.69807400	0.62935700
C	-2.28303400	3.85974400	1.76483300
C	-1.08510500	3.11511700	1.58092100
C	-0.51849200	3.49302200	0.33374400
H	-1.22540600	4.93277200	-1.22013600
H	-3.28772300	5.36533200	0.45432900
H	-2.95974100	3.77664200	2.60184500
H	-0.69506500	2.35720200	2.24397000
H	0.36871400	3.06601100	-0.11012600
C	-1.36587400	4.47177900	-0.25414500
Fe	-2.39691400	2.72690500	0.06000800

TS-mx
E = -3002.002885 a.u.
1 imaginary frequency -377.3525 cm-1

C	0.18604800	-2.39494900	0.78283200
N	-1.13440500	-2.46726400	0.97742500
C	-2.01348900	-2.26496000	-0.05357500
C	-1.17656000	-0.15101300	-0.51426000
C	0.10641700	-0.26223600	0.01630500
C	1.24708200	-0.32944500	-0.89590200
H	-1.26225900	-0.23017900	-1.59316800
H	0.31816900	0.14480300	0.99832900
O	1.15675600	-0.51031300	-2.10052300
C	-3.46394900	-2.12319500	0.01048400
C	-3.93567400	-2.32844300	-1.30387600
C	-5.26218800	-2.15402600	-1.65188000
C	-6.15713800	-1.77679700	-0.65354600
C	-5.71896100	-1.57545500	0.64901300
C	-4.37748700	-1.73756300	0.98523400
C	-1.69052600	-2.78515500	-1.40144000
H	-5.59431600	-2.31065800	-2.67192300
H	-4.07036200	-1.54449300	2.00314300
C	-1.49343500	-2.26338400	2.39220700
H	0.57604900	-2.74000000	-0.16282900
H	-2.01177700	-3.15540000	2.75281900
N	-2.87198600	-2.68590100	-2.11841800
H	-2.16022200	-1.40849500	2.48134200
O	-0.64578100	-3.22258600	-1.84168800
H	-2.93713000	-2.99437800	-3.07282700
H	-7.20376100	-1.63931000	-0.89949900
H	-6.42451000	-1.27599700	1.41475500
C	0.91124600	-2.48068600	2.08690600
H	1.22408700	-3.51516600	2.27239300
H	1.80122900	-1.85122600	2.09382700
C	2.58864500	-0.15200800	-0.28456800
N	4.69541000	0.00912600	0.09596200
N	4.05077700	0.20464200	1.27145400
C	3.81743600	-0.22601600	-0.91049200
C	4.18622300	-0.53110000	-2.31896500
H	5.13622200	-1.06657700	-2.37096600
H	3.38969900	-1.12164700	-2.76711700
H	4.27466400	0.38800500	-2.90470900
C	-0.15122300	-2.00887300	3.09043400
H	-0.08785100	-2.52114100	4.05019700
H	-0.02702400	-0.93960400	3.27413100
N	2.79164600	0.10956400	1.03760400
C	6.11734400	0.04117400	0.06650500
C	6.77855000	0.76183600	-0.92019300

C	6.82508100	-0.64357200	1.04712400
C	8.16693000	0.77788600	-0.93515300
H	6.21258300	1.31665300	-1.65788700
C	8.21178400	-0.60774500	1.03168500
H	6.28276300	-1.18359000	1.81229700
C	8.88474800	0.09489200	0.03850000
H	8.68659300	1.33728600	-1.70389700
H	8.76839700	-1.13692200	1.79600900
H	9.96811100	0.11444500	0.02686900
C	-3.42412000	1.86719800	1.68258900
C	-2.23553000	1.11011300	1.49286400
C	-2.22912600	0.61188400	0.15198800
C	-3.42812500	1.06802800	-0.46967600
H	-5.09473500	2.34616800	0.27923000
H	-3.69909900	2.39893600	2.58108100
H	-1.44662700	0.98536300	2.22058700
H	-3.71125700	0.86784000	-1.49241700
C	-4.15826000	1.84339400	0.46637700
C	-2.37367300	4.69504200	-0.04722100
C	-1.28690100	4.32537500	0.79229400
C	-0.42306800	3.47701000	0.04572400
C	-0.97544500	3.32227300	-1.25406500
H	-2.85086700	4.13471000	-2.15668700
H	-3.21319800	5.31191400	0.23587900
H	-1.15707600	4.61151400	1.82510800
H	0.47033800	2.99366200	0.41314100
H	-0.57240400	2.69848200	-2.03816500
C	-2.18122600	4.07440700	-1.31215600
Fe	-2.29311200	2.65437200	0.16299600

23

E = -3002.071765 a.u.

C	2.16335300	1.82256100	-0.28971500
N	0.91388500	2.57041000	-0.06731400
C	-0.19288900	1.69279900	-0.42853200
C	0.37783400	0.21177900	-0.35084400
C	1.82525600	0.37703800	0.12442300
C	-0.47148300	-0.68956600	0.51566700
H	1.83266500	0.31131100	1.21761300
H	0.36313100	-0.19418800	-1.36533100
O	-0.14796100	-1.03936100	1.63353500
C	-1.50939300	1.96098200	0.26983100
C	-2.45929300	2.32480200	-0.68963100
C	-3.78787600	2.53328800	-0.36165700
C	-4.16291800	2.37658600	0.97142200
C	-3.23043100	2.02892400	1.94064100
C	-1.89666600	1.81795500	1.58952300
C	-0.54998400	1.98770400	-1.91130600

H	-4.51395300	2.80657500	-1.11870100
H	-1.18179100	1.50804400	2.34172300
C	0.99070500	3.11547800	1.28585000
H	2.40585800	1.85551200	-1.35350100
H	0.85249300	2.35272600	2.07051600
N	-1.86381500	2.39152000	-1.94316300
H	0.24153400	3.89432600	1.43537100
O	0.18317400	1.88776300	-2.85969800
H	-2.35159300	2.53812600	-2.81077800
H	-5.19895000	2.52972700	1.25189700
H	-3.53934100	1.91376500	2.97267500
C	3.21122200	2.53791400	0.57825000
H	3.65489000	1.83025600	1.27989200
H	4.02088100	2.95707900	-0.02032900
C	-1.78861000	-1.03420200	-0.04365900
N	-3.88794100	-1.35693100	-0.31566200
N	-3.36514100	-1.00519500	-1.51879600
C	-2.93378300	-1.38563100	0.64206500
C	-3.17505300	-1.64713200	2.08384900
H	-3.16615900	-2.71953700	2.29552300
H	-2.37017600	-1.19431700	2.66056200
H	-4.13773800	-1.23872900	2.39734600
C	-5.29215000	-1.53464200	-0.18300200
C	-5.79744700	-2.66219100	0.45053400
C	-6.13840400	-0.56052300	-0.69800400
C	-7.17222800	-2.80696500	0.58238000
H	-5.12191200	-3.42338300	0.82113300
C	-7.51056900	-0.72263500	-0.57450700
H	-5.70840000	0.30322600	-1.18818300
C	-8.02883900	-1.84035800	0.07022500
H	-7.57328600	-3.68436000	1.07555200
H	-8.17741200	0.03013800	-0.97798300
H	-9.10123300	-1.96019100	0.16932800
C	2.42274000	3.63290100	1.32114100
H	2.78253100	3.80199500	2.33769200
H	2.47875500	4.57741900	0.77530300
N	-2.11101400	-0.80906000	-1.34786500
C	5.43220500	-0.28943900	1.54171700
C	5.92300800	0.32556200	0.35671700
C	6.61460500	-0.66132900	-0.39569100
C	6.55176200	-1.88578300	0.32290400
H	5.57305200	-2.39726000	2.26472100
H	4.84847200	0.18637100	2.31517200
H	5.77748100	1.35384100	0.06595600
H	7.07664000	-0.51384200	-1.36002900
H	6.95484200	-2.83313600	-0.00192900
C	5.82079700	-1.65618500	1.51998900
C	2.68960200	-0.71514900	-0.42618600
C	3.40128100	-0.72515600	-1.66044200
C	3.94032800	-2.02551200	-1.85467800

C	3.56045300	-2.82897100	-0.74594900
H	2.35966300	-2.32351900	1.07653500
H	3.52663200	0.11419900	-2.32752500
H	4.56124800	-2.33570300	-2.68160500
H	3.84307900	-3.85778300	-0.58055800
C	2.79157100	-2.02176300	0.13372200
Fe	4.66124400	-1.22422900	-0.11544000

24
E = -3002.062666 a.u.

C	2.08064700	1.66758400	0.99633300
N	0.93239500	2.45631300	0.50362400
C	-0.22072100	1.56726400	0.53397700
C	0.35199100	0.16850500	0.09143200
C	1.80686000	0.17169200	0.59462300
C	-0.48539900	-0.98671500	0.59931800
H	1.82783300	-0.41559400	1.51460600
H	0.31354000	0.14577600	-1.00188900
O	-0.16077600	-1.68929800	1.53187000
C	-1.44890200	2.03314800	-0.21484200
C	-2.51185100	2.15232500	0.68222800
C	-3.78676200	2.50189000	0.27614200
C	-3.98561400	2.75040000	-1.08109400
C	-2.94505200	2.62412600	-1.99131400
C	-1.67274700	2.25179800	-1.56028800
C	-0.73889500	1.51333000	2.00142900
H	-4.60207600	2.58112500	0.98584400
H	-0.87951600	2.10966300	-2.28282400
C	1.33034100	2.95451300	-0.81337100
H	2.06375000	1.69390100	2.08378600
H	0.66688900	3.75648300	-1.14006200
N	-2.07713200	1.82691600	1.96693900
H	1.31828900	2.16406100	-1.58419200
O	-0.11502700	1.24259100	2.99557500
H	-2.62286900	1.91131100	2.80822200
H	-4.97184900	3.03886700	-1.42799300
H	-3.12529600	2.79520300	-3.04522900
C	3.31326000	2.40767400	0.45546800
H	3.83171300	2.92176400	1.26641500
H	4.02177500	1.71404500	0.01314200
C	-1.80682900	-1.14009200	-0.03789600
N	-3.90622300	-1.33532400	-0.40806700
N	-3.31480700	-0.94676700	-1.56655800
C	-2.99114200	-1.47476500	0.58137000
C	-3.28589500	-1.81455800	1.99792500
H	-3.35177200	-2.89629800	2.13778000
H	-2.47033100	-1.45787600	2.62647800
H	-4.22858700	-1.36766000	2.32102900

C	-5.32258500	-1.43278100	-0.34651800
C	-5.92617700	-2.52183700	0.26908200
C	-6.08287400	-0.41991400	-0.91954800
C	-7.31227400	-2.58540300	0.32792800
H	-5.31909900	-3.31830500	0.68107200
C	-7.46665100	-0.50254100	-0.86860300
H	-5.57695600	0.40832400	-1.39899100
C	-8.08325600	-1.57870800	-0.23974500
H	-7.78879400	-3.43225000	0.80725000
H	-8.06598700	0.28068300	-1.31768900
H	-9.16456800	-1.63573500	-0.19766000
C	2.76279200	3.41702600	-0.57336400
H	2.75735000	4.42504700	-0.15395400
H	3.34418400	3.44261300	-1.49795700
N	-2.06113200	-0.82150700	-1.33725400
C	6.68708900	-1.02722500	-0.56109600
C	6.29056800	-2.13554000	0.23635700
C	5.57229400	-1.63674700	1.35738900
C	5.52434400	-0.22061500	1.25389700
H	6.33836200	1.16353500	-0.30150600
H	7.23061400	-1.07727700	-1.49240300
H	6.47597200	-3.17558500	0.01444800
H	5.11546800	-2.23051200	2.13448000
H	5.02799000	0.44796100	1.94109500
C	6.21336400	0.15645800	0.06740100
C	3.54019200	-2.38128400	-1.52496300
C	2.80787500	-1.97649200	-0.37907500
C	2.66531100	-0.55557400	-0.40165300
C	3.30573800	-0.09484100	-1.59201000
H	4.43034300	-1.17996400	-3.18536900
H	3.83766800	-3.39064500	-1.76594200
H	2.42474400	-2.61776500	0.40131500
H	3.38941600	0.93031100	-1.91953200
C	3.85388500	-1.21557300	-2.27316700
Fe	4.64419100	-1.08608200	-0.38525500

25

E = -3002.074949 a.u.

C	1.04185900	-2.52344200	1.00162300
N	2.42323500	-2.56407600	0.49118900
C	2.88075300	-1.18398400	0.42196800
C	1.64051300	-0.35953500	-0.03479200
C	0.45249700	-1.11611200	0.57355800
C	-0.67292900	-1.22911600	-0.42658300
H	1.58434700	-0.47736800	-1.12052900
H	0.07839200	-0.62220000	1.46763100
O	-0.47900500	-1.40396100	-1.61728800
C	4.11594600	-0.90342600	-0.39624000

C	5.10506100	-0.37883900	0.43785600
C	6.33149600	0.03585300	-0.04871700
C	6.55678600	-0.07879200	-1.41943600
C	5.57786600	-0.57984100	-2.26777500
C	4.34458900	-0.98628500	-1.75598100
C	3.33506800	-0.77036500	1.84838500
H	7.08901100	0.44447600	0.60974200
H	3.57516400	-1.35930900	-2.42190300
C	2.39198900	-3.33399000	-0.75276800
H	1.06782400	-2.51847800	2.08894100
H	3.39824300	-3.64969900	-1.03338800
N	4.63194900	-0.33273000	1.74795700
H	1.94919300	-2.77306900	-1.59173500
O	2.68969500	-0.82074200	2.86649600
H	5.13931800	0.01954300	2.54206800
H	7.51003400	0.23802500	-1.82709400
H	5.76947200	-0.65074500	-3.33170800
C	0.38068200	-3.80997600	0.46371900
H	0.03627200	-4.44333600	1.28187300
H	-0.48749000	-3.59419500	-0.16013800
C	-2.04420700	-1.11765100	0.08835100
N	-4.15734700	-0.84716800	0.32607000
N	-3.59277800	-0.81028200	1.56249800
C	-3.22237000	-1.03480200	-0.63182800
C	-3.49328800	-1.06347600	-2.09295000
H	-4.30954600	-0.38670400	-2.35207000
H	-2.58698500	-0.78470100	-2.62640600
H	-3.76432400	-2.07165500	-2.41790700
C	1.47794100	-4.49093200	-0.37459700
H	2.03310700	-5.20899700	0.23350900
H	1.08104800	-5.01196800	-1.24760700
N	-2.33114700	-0.97086800	1.41310000
C	-5.55857700	-0.63796200	0.18819400
C	-6.31954000	-1.50902100	-0.58061100
C	-6.14194400	0.43995400	0.84216900
C	-7.68359900	-1.28445200	-0.70983500
H	-5.85160200	-2.36093400	-1.05829100
C	-7.50822000	0.64604900	0.71798500
H	-5.52364400	1.09557600	1.44161700
C	-8.27866200	-0.20931700	-0.06162700
H	-8.28279500	-1.95956100	-1.30903200
H	-7.97074700	1.48298300	1.22728500
H	-9.34440600	-0.04018900	-0.16032000
C	2.40064700	3.30817200	0.06182300
C	2.35180500	2.05618700	-0.60542900
C	1.75785900	1.10080300	0.27000800
C	1.44751700	1.77419000	1.48789800
H	1.70236900	3.90442300	2.09898300
H	2.76278700	4.23615100	-0.35442400
H	2.68732400	1.85213400	-1.61176800

H	0.98709800	1.32679900	2.35519200
C	1.84143900	3.13256500	1.35698100
C	-0.68040800	4.22453200	-0.73699000
C	-0.55552600	3.24383700	-1.75842500
C	-1.08815800	2.02226400	-1.26328500
C	-1.54232600	2.24806300	0.06665400
H	-1.49272700	4.08090200	1.33931600
H	-0.34029100	5.24754100	-0.79430100
H	-0.10532300	3.39091700	-2.72845700
H	-1.10352700	1.08394900	-1.79585000
H	-1.96663400	1.51370000	0.73366700
C	-1.28992800	3.60897100	0.38986400
Fe	0.46386000	2.65495100	-0.07889500

26

E = -3002.079989 a.u.

C	-1.80155700	2.52118100	1.18565000
N	-3.16197300	1.96589200	1.09672200
C	-3.15165800	0.63798700	0.53671300
C	-1.69912100	0.15623900	0.83782400
C	-0.88825800	1.44365500	0.58812300
C	0.46670000	1.40258800	1.25678200
H	-1.67036700	-0.05964100	1.91092700
H	-0.75875800	1.59869700	-0.48388400
O	0.57721800	1.37026100	2.46711400
C	-3.55402600	0.47493100	-0.91968400
C	-4.57198100	-0.47454900	-1.00259600
C	-5.11397100	-0.86804400	-2.21250000
C	-4.61575000	-0.27153300	-3.36952600
C	-3.61456800	0.68883900	-3.30649300
C	-3.08053600	1.06619000	-2.07417500
C	-4.14333000	-0.31967600	1.25471600
H	-5.89778100	-1.61503600	-2.26228700
H	-2.29883400	1.81623000	-2.03028500
C	-4.10815800	2.97322400	0.63302600
H	-1.53000000	2.66474300	2.23767200
H	-5.01787300	2.97267100	1.24115900
N	-4.90858000	-0.91389200	0.27975100
H	-4.40327600	2.80220000	-0.41136000
O	-4.21094200	-0.53787700	2.43666500
H	-5.60077900	-1.61201700	0.49271700
H	-5.02139400	-0.56253700	-4.33181300
H	-3.24428000	1.14447400	-4.21692700
C	-1.88541600	3.87021800	0.47348000
H	-1.14750200	4.58998000	0.83308500
H	-1.73632300	3.73852000	-0.60418400
C	1.65667800	1.37420600	0.39501200
N	3.63923400	1.19892500	-0.39964800

N	2.78136000	1.30380500	-1.44907500
C	2.98019400	1.24891600	0.77844400
C	3.62342800	1.17595900	2.11638500
H	4.62004200	1.62041000	2.09755900
H	2.99428600	1.68675100	2.84225300
H	3.71579700	0.13429500	2.43686000
C	-3.33144600	4.29031100	0.75792500
H	-3.41145400	4.67966900	1.77718000
H	-3.70154500	5.05628400	0.07433200
N	1.59955200	1.40976400	-0.96570300
C	5.03168700	1.02399000	-0.64177700
C	5.70100300	-0.04100300	-0.05223700
C	5.68733500	1.91028000	-1.48585300
C	7.05789400	-0.20441300	-0.29672500
H	5.16091600	-0.74167200	0.57366900
C	7.04048800	1.72859000	-1.73378200
H	5.13257900	2.71978400	-1.94285700
C	7.72751700	0.67825800	-1.13540500
H	7.58767700	-1.03195400	0.15956600
H	7.56031400	2.41311300	-2.39335900
H	8.78532400	0.54393900	-1.32833900
C	-0.68186400	-2.48558800	-1.63693800
C	-0.61978500	-1.14641000	-1.17157000
C	-1.33170800	-1.06805300	0.05898600
C	-1.83411800	-2.37319100	0.34631900
H	-1.63670500	-4.30413900	-0.75678300
H	-0.20635300	-2.86587900	-2.52833400
H	-0.09482200	-0.33289000	-1.64976500
H	-2.40222900	-2.64169100	1.22533600
C	-1.43764900	-3.24436900	-0.70259200
C	1.22779200	-4.00336600	1.03169100
C	2.00605300	-3.38217400	0.01714100
C	2.20038500	-2.02176200	0.38277800
C	1.54532300	-1.80445800	1.62964900
H	0.35082000	-3.18039300	2.91563000
H	0.88521600	-5.02711400	1.03048900
H	2.35622100	-3.84938800	-0.89085400
H	2.71398100	-1.28417700	-0.21480200
H	1.47844200	-0.87687600	2.17621400
C	0.94424100	-3.02889000	2.02684000
Fe	0.20270700	-2.42982300	0.21011000
